# Understanding the Mahaim Pathway in the Context of Catheter Ablation

**DOI:** 10.19102/icrm.2019.100104

**Published:** 2019-01-15

**Keywords:** Atrioventricular node, catheter ablation, Mahaim pathway

## Dr. Smeets discusses

Historically, it was suggested by Ivan Mahaim in 1938^1^ that conducting fibers may exist from the atrioventricular (AV) node (nodoventricular fibers) to the ventricle. Other fibers from the His bundle and fascicle to the right ventricle (RV) (fasiculoventricular fibers) and from the AV node to the right bundle (nodofascicular fibers) were subsequently defined later.^2^

Creating AV block with direct-current energy^3^ or with cryo energy^4^ revealed, however, total AV block with preserved preexcitation due to conduction over an accessory pathway (AP) connecting the right atrium with the RV. These decremental anterograde-conducting connections, not localized in the AV node or the distal part of the conduction system, were mentioned as being Mahaim-like APs. However, more in detail, the definition of these fibers is as follows: decrementally conducting accessory AV pathways connecting the right atrium with a part of the right bundle branch (RBB), or atriofascicular (AF) pathways.

These decrementally conducting APs (which can be either short or long in length) may have their atrial origins all along the border of the “saddle”-shaped tricuspid valve (TV). Catheter mapping of the TV is very challenging because of its oval shape, which changes further during RV contraction. Notably, the TV is not located in a single horizontal plane like the mitral valve; rather, the anteroseptal part of the TV is superior and the posteroseptal part is inferior, respectively, in relation to the major part of the oval body of the TV.

In light of this information, one question to consider is: how can one localize the decremental AP in Mahaim-related tachycardias?

### Atrial insertion of atriofascicular connection

My first approach strategy is to try to localize the atrial origin of the pathway. In fast-conducting pathways (Kent bundles)—which are, in general, short in length—the shortest A–V time provides key information about the atrial and the ventricular insertions of the AP. Sometimes, the anatomy can be complicated, like in the posteroseptal region of the heart, since the AP may cross from the right atrium to the summit of the left ventricle and from the left atrium to the summit of the RV. In general, the atrial and ventricular connections of the AP are quite close to one another in the vertical plane. In contrast, the AF pathways may be short, though they can also be very long, extending deep into the RV and connecting to the right bundle or its branches.

The most helpful criterion to localize the atrial insertion of the AF pathway is the presence of the Mahaim potential (M potential). The best circumstance to delineate the M potential is during atrial pacing or antidromic tachycardia (AT). However, the M potential is not always easy to locate along the TV border due to its complex anatomy (see above) and dynamic behavior present during RV contraction. One should realize that the M potential can be very sensitive for catheter manipulation and can be (for a prolonged time!) blocked by mechanical force. Sometimes (in certain reports, even up to 50% of the time^5^), the M potential cannot be localized.

**Figure 1:**
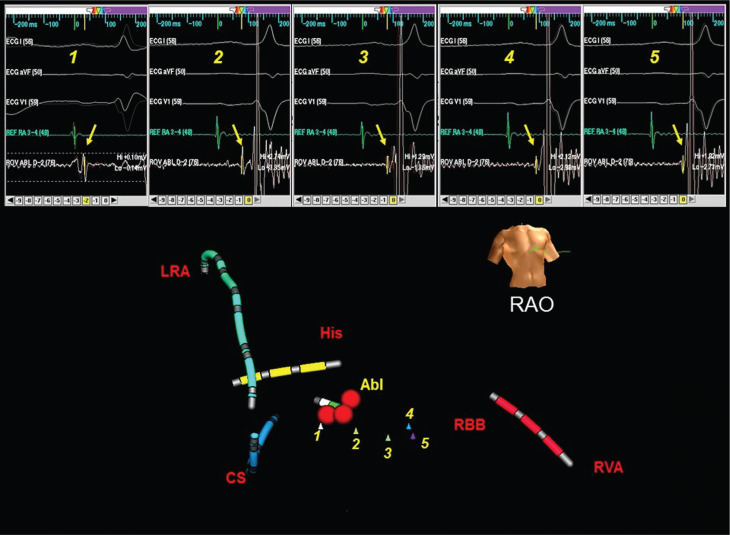
The anatomic course of the AP inserting near the RBB is outlined using an electroanatomic mapping system. Shown on the map are the locations of accessory pathway potentials (small triangles, labeled 1 to 5), with the corresponding electrograms are recorded above (arrows, labeled 1 to 5). The red balls indicate the site at which ablation was performed, specifically the atrial insertion site on the tricuspid annulus. Reproduced with permission from: Gupta A, Hsia HH, Po R, Zei PC. Electroanatomic localization of a slowly conducting atrioventricular (Mahaim) accessory pathway. *J Interv Card Electrophysiol.* 2011;31(2):119–124.

In the elegant case report by Lokhandwala et al.,^6^ the M potential could not be detected. However, an AP, not included in the proximal part of the AV conduction system, should be present, as nicely illustrated in the authors’ **Figure 1**.

### Ventricular insertion of atriofascicular connection

In contrast to in the case of Wolff–Parkinson–White syndrome, the determination of the QRS axis during maximal preexcitation (ie, AT) does not indicate the ventricular insertion of the AP and, as such, is not helpful in determining the optimal ventricular site for catheter ablation.^7^ One of the explanations for this is the fact that the length of the AF pathways may vary from short to long and the site of insertion in the conduction system/right bundle may be different, resulting in disparate widths of the QRS complex.

To localize the ventricular insertion of the AF pathway, the RV must be mapped extensively, which can be very time-consuming to do. The earliest ventricular activation preceding the V signal on the 12-lead electrocardiogram should be the target to look for. This earliest ventricular signal should preferably be preceded by a sharp deflection (conducting system, RBB). In Lokhandwala et al.’s **Figure 4**,^6^ the distal radiofrequency signal in the preexcited complexes demonstrates a sharp deflection from the distal conduction system followed by immediate ventricular activation. In contrast, in the nonpreexcited complex, the QRS complex seems to be somewhat later relative to the spike of the conduction system, indicating a slight change in activation locally in the absence/connection of the AF fiber. Depending on the site of radiofrequency application, RBB conduction may change or even lead to RBB block.

The last wide QRS complex is not preceded by a signal from the conduction system (no spike visible) and is most likely due to a ventricular premature beat.

Joep L. R. M. Smeets, MD (jsmeets58@gmail.com)^1^

^1^Radboud University Medical Center, Radboud University, Nijmegen, the Netherlands

The author reports no conflicts of interest for the published content.

References1.Benatt MahaimINouvelles recherches sur les connexions superiors de la branche gauche du faisceau de His-Tawara avec cloison interventriculaire.Cardiologia.19381611202.AndersonRHBeckerAEBrechenmacherCDaviesMJRossiLVentricular preexcitation. A proposed nomenclature for its substrates.Eur J Cardiol.1975312736[PubMed]11324073.BhandariAMoradyFShenENCatheter induced His Bundle ablation in a patient with reentrant tachycardia associated with a nodoventricular tract.J Am Coll Cardiol.198443611616[CrossRef][PubMed]647034310.1016/s0735-1097(84)80109-64.KleinGJGuiraudonGMKerrCR“Nodoventricular” accessory pathway: evidence for a distinct accessory pathway with atrioventricular node-like properties.J Am Coll Cardiol.198811510351040[CrossRef][PubMed]312858610.1016/s0735-1097(98)90063-85.KothariSGuptaAKLokhandwalaYYVoraAKerkarPGThakurRKAtriofascicular pathways: where to ablate?Pacing Clin Electrophysiol.2006291112261233[CrossRef][PubMed]1710067510.1111/j.1540-8159.2006.00527.x6.LokhandwalaYVyasAA wide complex tachycardia in a structurally normal heart: what is the mechanism? Where to ablate?J Innov Cardiac Rhythm Manage.20191013504350710.19102/icrm.2018.091202PMC7252864324944057.SternickEBLokhandwalaYBohoraSIs the 12-lead electrocardiogram during antidromic circus movement tachycardia helpful in predicting the ablation site in atriofascicular pathways?Europace.2014161116101618[CrossRef][PubMed]2468176210.1093/europace/euu059

## Drs. Hsia and Sanchez comment

The case by Lokhandwala and Vyas^1^ nicely demonstrates some classic electrophysiology findings of an anterograde slowly conducting AF (“Mahaim”) AP with successful catheter ablation.^2,3^ This case study notably highlights the importance of alternative target sites for ablation when the AP potential cannot be identified along the tricuspid annulus.

Lokhandwala et al.’s **Figure 1B** shows the termination of tachycardia in response to an atrial premature stimulus. Although the atrial signals on the coronary sinus catheter remain on-time, septal atrial activation (His bundle and right bundle electrograms) is clearly advanced. This does not rule out AV nodal reentrant tachycardia (AVNRT) with a bystander Mahaim conduction. However, a late right-free-wall atrial premature delivery that occurred after the septal A was committed resulted in a resetting of the tachycardia (both V and A) (**Figure 1A** by Lokhandwala et al.). This conclusively excludes the possibility of AVNRT, since such late ventricular depolarization cannot advance the subsequent atrial activation in AVNRT, thereby confirming AV reentrant tachycardia using the AF pathway as the antegrade limb of the reentrant circuit.

The anatomical variants of so-called “Mahaim” pathways include AF and AV insertions that can have either short or long connections.^4,5^ The atrial insertions of Mahaim pathways are often located at the right anterolateral to posterolateral TV, and catheter ablation is highly effective when targeting AP potential.^4^ However, AP potentials along the TV annulus may not be identified in a significant minority of patients.^6^ In the present report, the Mahaim potential could not recorded when mapping the lateral TV annulus, and targeting the earliest local ventricular activation successfully eliminated preexcitation (**Figure 4** by Lokhandwala et al.).

AP potential can also be identified along the course of the Mahaim fiber between the TV annulus and the RV insertion sites, either on the free wall or near the septal right bundle **(Figure 1)**.^4,5,7^ Ablation of the Mahaim pathway targeting the distal insertion was first reported during atrial fibrillation.^8^ However, the distal insertion of the Mahaim fiber may be highly arborized over a wide area with diffuse activation, and unsuccessful ablation is often associated with a change in the preexcitation pattern.^5^ It is, therefore, important not only to map the earliest local ventricular activation but also to identify the precise fascicular/ventricular insertion sites by way of the high-frequency presystolic AP potential recordings (**Figure 4**, first two beats, by Lokhandwala et al.). Despite this, mapping and ablation of the Mahaim fibers guided by careful mapping of AP potentials along the TV annulus is still preferable, as the annular target site is more discrete and localized in this instance.

Henry H. Hsia, MD, FHRS (henry.hsia@ucsf.edu)^1^ and Jose M. Sanchez, MD^1^

^1^University of California, San Francisco, San Francisco, CA, USA

The authors report no conflicts of interest for the published content.

References1.LokhandwalaYVyasAA wide complex tachycardia in a structurally normal heart: what is the mechanism? Where to ablate?J Innov Cardiac Rhythm Manage.20191013504350710.19102/icrm.2018.091202PMC7252864324944052.LüderitzBIvan Mahaim (1897–1965).J Interv Card Electrophysiol.200382155[CrossRef][PubMed]1276650810.1023/a:10236171190933.KatritsisDGWellensHJJosephsonMEMahaim accessory pathways.Arrhythm Electrophysiol Rev.2017612932[CrossRef][PubMed]2850774410.15420/aer.2016:35:1PMC54309434.McClellandJHWangXBeckmanKJRadiofrequency catheter ablation of right atriofascicular (Mahaim) accessory pathways guided by accessory pathway activation potentials.Circulation.199489626552666[PubMed]820567810.1161/01.cir.89.6.26555.HaïssaguerreMCauchemezBMarcusFCharacteristics of the ventricular insertion sites of accessory pathways with anterograde decremental conduction properties.Circulation.199591410771085[PubMed]785094410.1161/01.cir.91.4.10776.KothariSGuptaAKLokhandwalaYYVoraAMKerkarPGThakurRKAtriofascicular pathways: Where to ablate?Pacing Clin Electrophysiol.2006291112261233[CrossRef][PubMed]1710067510.1111/j.1540-8159.2006.00527.x7.GuptaAHsiaHHLoRZeiPCElectroanatomic localization of a slowly conductiong atrioventricular (Mahaim) accessory pathway.J Interv Card Electrophysiol.2011312119124[CrossRef][PubMed]1994309910.1007/s10840-009-9440-58.MillerJMHarperGRRothmanSAHsiaHHRadiofrequency catheter ablation of am atriofascicular pathway during atrial fibrillation: a case report.J Cardiovasc Electrophysiol.1994510846853[CrossRef][PubMed]787433010.1111/j.1540-8167.1994.tb01123.x

## Drs. Lerman and Markowitz remark

The case by Lokhandwala and Vyas^1^ presents an interesting dilemma. A Mahaim potential was not targeted for ablation since it was not observed in the tricuspid annular (TA) region. How, then, should one approach ablating an AF pathway when the optimal choice is not available? The approach under such circumstances is dictated by the anatomic course of the pathway. An AF pathway originates in the atrial tissue bordering the anterolateral to posterolateral tricuspid annulus, traverses the TA region, and inserts into the distal RBB in the region of the apical third RV free wall along the course of the moderator band or its insertion into the anterior papillary muscle. Therefore, it may be useful to consider approaching the pathway from any of three anatomic sites, as follows.

### Atrial insertion

One option is to atrially pace circumferentially adjacent to the TA annulus, concentrating on the anterior/lateral regions and determining the site that produces maximal preexcitation and the shortest AV interval. This presumably would be the site of the atrial insertion and would be an appropriate ablation target. It has also been suggested that ablating from the ventricular aspect of the tricuspid annulus, directly opposite the optimal atrial pacing site, can be an effective approach, even though it doesn’t correspond to the earliest site of ventricular activation. A more cumbersome option would be mapping and ablating from the site that marks the longest atrial extrastimulus coupling interval that resets preexcited antidromic reciprocating tachycardia during septal atrial refractoriness.

### Tricuspid annulus

AF pathways are particularly susceptible to temporary catheter bump suppression. These pathways may take several hours to resume conduction. If the site of mechanical abolition of AF conduction is annotated on an electroanatomical mapping system, empiric ablation could be performed at this site, albeit with a relatively low probability of long-term success. Although neither optimal nor elegant, if preexcitation is absent for any reason, a series of ablation applications could be made along the most likely TA course of the pathway (anterior/lateral).

### Ventricular insertion

The approach taken by Lokhandwala and Vyas in their case study^1^ was an appropriate and practical option, since the Mahaim potential was not identified. They mapped the site of earliest ventricular activation relative to QRS onset during atrial pacing, which is presumably near the distal insertion site of the pathway. A variation of this approach would be to map the RV free wall, identifying early Purkinje potentials (in the region of the moderator band–anterior papillary muscle interface), which correspond to the insertion site of the AF pathway into the distal RBB. These potentials likely originate from the distal RBB and precede earliest ventricular activation. Intracardiac echocardiography (ICE) may help to facilitate positioning of the ablation catheter in the region of the moderator band and anterior papillary muscle. Alternative approaches include ventricular pace-mapping and identification of the site with the best QRS match as well as entraining during antidromic reciprocating tachycardia from this site and confirming that the postpacing interval is equivalent to the tachycardia cycle length. There are several caveats to be mindful of when ablating at the distal insertion site. First, the distal end of the pathway may arborize, so the placement of a discrete lesion at a single site may not be successful. Furthermore, damaging but not completely eliminating the distal insertion site may result in damage to the RBB, giving rise to a subsequent slower and incessant tachycardia, due to retrograde conduction over the left bundle. To maximize catheter stability, ablation should be performed during atrial pacing.

Bruce B. Lerman, MD (blerman@med.cornell.edu)^1^ and Steven M. Markowitz, MD^1^

^1^Department of Medicine, Division of Cardiology, Cornell University Medical Center, New York, NY, USA

The authors report no conflicts of interest for the published content.

Reference1.LokhandwalaYVyasAA wide complex tachycardia in a structurally normal heart: what is the mechanism? Where to ablate?J Innov Cardiac Rhythm Manage.20191013504350710.19102/icrm.2018.091202PMC725286432494405

## Dr. Van Hare reminds

Drs. Lokhandwala and Vyas^1^ have presented a nicely documented case of Mahaim antidromic tachycardia with successful ablation, despite not being able to record a discrete Mahaim potential on the tricuspid annulus. I’d like to start by pointing out that working with Mahaim pathways is fun! When I was a fellow training at the University of California, San Francisco, we could always get Mel Scheinman excited by simply asking “could this be a Mahaim?” with regard to any wide QRS tachycardia, and this would lead to a wonderful five- or 10-minute teaching session on the evaluation of wide complex tachycardias and maneuvers to prove Mahaim pathways.

The case presented demonstrates nearly all of the classic criteria for Mahaim AF pathways. Just to add, the maneuver of introducing premature atrial beats (PACs) into antidromic tachycardia, at a time at which the low septal atrial electrogram is committed, proves that the pathway is AF rather than nodofascicular, which is a useful piece of information if a Mahaim potential is not found on the tricuspid annulus. Termination by the PACs without advancing the ventricle excludes ventricular tachycardia, which is also a useful maneuver. Notably, in their report, the authors wonder whether or not there are other maneuvers for mapping a Mahaim pathway. One of the oldest techniques is to pace on the atrial side of the tricuspid annulus at various sites around the ring and measure stimulus-to-V times; the shortest should get you close.^2^ If one observes a sudden loss of preexcitation with this maneuver, then you’ve found it (“bump mapping”). If you don’t bump it, then I would expect that the delivery of radiofrequency energy at the candidate site would cause Mahaim automaticity, essentially an irregular wide complex accelerated rhythm with the same morphology as that of a fully preexcited QRS, and then a sudden loss of preexcitation. I have found Mahaim automaticity to be one of the most helpful observations when performing ablations in this substrate.

George F. Van Hare, MD^1^

^1^Department of Pediatrics, Washington University School of Medicine, St. Louis, MO, USA

The author reports no conflicts of interest for the published content.

References1.LokhandwalaYVyasAA wide complex tachycardia in a structurally normal heart: what is the mechanism? Where to ablate?J Innov Cardiac Rhythm Manage.20191013504350710.19102/icrm.2018.091202PMC7252864324944052.KleinLSHackettFKZipesDPMilesWMRadiofrequency catheter ablation of Mahaim fibers at the tricuspid annulus.Circulation.1993873738747[PubMed]844389410.1161/01.cir.87.3.738

## Dr. Han shares

Mapping and ablation of an AF pathway participating in an antidromic tachycardia is typically focused on a putative pathway potential along the tricuspid annulus or on the RV free wall at the insertion into the RBB.

Several factors can make the mapping and ablation of these pathways challenging, as follows: (1) the location along the lateral tricuspid annulus and RV free wall, (2) the concern of “bumping the pathway” and losing the pathway potential, (3) the concern of perforation with placement of the ablation catheter along the RV free wall, (4) suboptimal catheter contact and stability, (5) the concern of causing a RBBB or an incessant tachycardia with ablation at the distal insertion of the pathway into the RV, and (6) the potential for varying lengths of the AF pathway.

In Lokhandwala and Vyas’s report of distal ablation for an AF pathway,^1^ the authors describe a case of an AF pathway where they could not identify a pathway potential along the tricuspid annulus and thus performed mapping for the earliest ventricular electrogram, which was mapped to the apical RV. The authors were able to successfully ablate the pathway at this location consistent with the presence of a long AF pathway with insertion into the apical ventricle.

For suspected AF pathways, I typically utilize a duodecapolar catheter with placement of the catheter arranged to record activation along the lateral tricuspid annulus, the cavotricuspid isthmus, and the proximal coronary sinus. This ensures coverage along the inferior, inferolateral, lateral, and superior tricuspid annulus in order to bracket the earliest ventricular activation and pathway potential along the tricuspid annulus. To maximize catheter stability, I typically use an Agilis™ medium or long curl sheath (Abbott Laboratories, Chicago, IL, USA) with a contact force catheter and ICE guidance. This provides me with greater confidence with regard to catheter stability at the location of interest as well as the ability to monitor for the potential of a perforation/pericardial effusion with the ablation catheter along the lateral tricuspid annulus and the RV free wall. By using contact force, one can also try to minimize mechanical trauma to a pathway location that may result in loss of antegrade preexcitation via the pathway. Finally, with mapping the RV fascicular system, which has branches along the moderator band, ICE is very useful for ensuring catheter stability during mapping and ablation.

As Sternick et al. showed,^2^ the correct AF pathway location cannot be identified reliably using the 12-lead electrocardiogram. As a result, my practice has been to focus on the mapping of an AF pathway potential along the tricuspid annulus. If a pathway potential cannot be identified along the tricuspid annulus, I then focus on mapping the earliest ventricular activation along the RV free wall. Since these pathways typically insert into the apical third of the RV, I start with mapping along the moderator band of the RV, where I can identify a right bundle potential and proceed with mapping towards the RV free wall to identify the earliest AF pathway potential into the right-sided conduction system. This helps to avoid prolonged catheter manipulation along the RV free wall and enables one to focus mapping and ablation efforts on the potential insertion site of the pathway.

Frederick T. Han, MD, FACC, FHRS (fredhan@ucsd.edu)^1^

^1^Section of Cardiac Electrophysiology, Division of Cardiovascular Medicine, University of California, San Diego, La Jolla, CA, USA

The author reports no conflicts of interest for the published content.

References1.LokhandwalaYVyasAA wide complex tachycardia in a structurally normal heart: what is the mechanism? Where to ablate?J Innov Cardiac Rhythm Manage.20191013504350710.19102/icrm.2018.091202PMC7252864324944052.SternickEBLokhandwalaYBohoraSIs the 12-lead electrocardiogram during antidromic circus movement tachycardia helpful in predicting the ablation site in atriofascicular pathways?Europace.2014161116101618[CrossRef][PubMed]2468176210.1093/europace/euu059
